# A Graph Fourier Transform Based Bidirectional Long Short-Term Memory Neural Network for Electrophysiological Source Imaging

**DOI:** 10.3389/fnins.2022.867466

**Published:** 2022-04-13

**Authors:** Meng Jiao, Guihong Wan, Yaxin Guo, Dongqing Wang, Hang Liu, Jing Xiang, Feng Liu

**Affiliations:** ^1^School of Systems and Enterprises, Stevens Institute of Technology, Hoboken, NJ, United States; ^2^College of Electrical Engineering, Qingdao University, Qingdao, China; ^3^Department of Dermatology, Massachusetts General Hospital, Harvard Medical School, Boston, MA, United States; ^4^Department of Biomedical Informatics, Harvard Medical School, Boston, MA, United States; ^5^Department of Electrical and Computer Engineering, Stevens Institute of Technology, Hoboken, NJ, United States; ^6^MEG Center, Division of Neurology, Cincinnati Children’s Hospital Medical Center, Cincinnati, OH, United States

**Keywords:** electroencephalography, source localization, inverse problem, graph Fourier transform, BiLSTM

## Abstract

Electrophysiological source imaging (ESI) refers to the process of reconstructing underlying activated sources on the cortex given the brain signal measured by Electroencephalography (EEG) or Magnetoencephalography (MEG). Due to the ill-posed nature of ESI, solving ESI requires the design of neurophysiologically plausible regularization or priors to guarantee a unique solution. Recovering focally extended sources is more challenging, and traditionally uses a total variation regularization to promote spatial continuity of the activated sources. In this paper, we propose to use graph Fourier transform (GFT) based bidirectional long-short term memory (BiLSTM) neural network to solve the ESI problem. The GFT delineates the 3D source space into spatially high, medium and low frequency subspaces spanned by corresponding eigenvectors. The low frequency components can naturally serve as a spatially low-band pass filter to reconstruct extended areas of source activation. The BiLSTM is adopted to learn the mapping relationship between the projection of low-frequency graph space and the recorded EEG. Numerical results show the proposed GFT-BiLSTM outperforms other benchmark algorithms in synthetic data under varied signal-to-noise ratios (SNRs). Real data experiments also demonstrate its capability of localizing the epileptogenic zone of epilepsy patients with good accuracy.

## Introduction

EEG/MEG source imaging (ESI), also known as EEG/MEG source localization, is a non-invasive neuroimaging technology that infers the location, direction, and distribution of the corresponding brain sources from the EEG or MEG data (He et al., 2018). Compared with the invasive modalities, the recording of EEG/MEG signals imposes minimum risks of blooding and inflammation of the brain (Portillo-Lara et al., 2021). Compared to other non-invasive brain imaging modalities, like computed tomography (CT), positron emission tomography (PET), functional magnetic resonance imaging (fMRI), and functional near-infrared spectroscopy (fNIRS), the temporal resolution of EEG is up to a millisecond (He et al., 2018), which allows it to track the electrical activity of neurons in smaller temporal granularity (Numata et al., 2019). The study of ESI is of great significance in both neuroscience and clinical applications (Congedo and Sherlin, 2011). Accurate estimation of brain sources can not only help neuroscientists to better understand the brain mechanism (Liu et al., 2019) and the pathological characteristics of brain injury or mental disorders (da Silva, 2013), but also help doctors to identify the lesion areas of brain diseases such as epilepsy focal regions, which can contribute to the improvement of the accuracy of presurgical evaluations (Sanei and Chambers, 2013).

However, the inverse problem of ESI is highly ill-posed (Qin et al., 2017; He et al., 2018; Cui et al., 2019), and there can be infinite numbers of source configurations that explain the EEG recording since the number of EEG sensors on the scalp is far less than the number of brain sources (Liu et al., 2017; Hecker et al., 2021). Consequently, numerous methods have been proposed to solve the ESI problem by incorporating different regularizations or prior information to seek a unique solution, as further discussed in see section “Related Work.” In recent years, deep learning has achieved great success in the fields of computer vision (Voulodimos et al., 2018), natural language processing (Young et al., 2018), bioinformatics (Min et al., 2017), etc., by employing its end-to-end feature extraction and representation capability (Deng and Yu, 2014; LeCun et al., 2015). Solving the inverse problems in the computer vision domain such as image reconstruction (Schlemper et al., 2017), super resolution (Dong et al., 2015), etc., has achieved great success by using a variety of artificial neural network (ANN) architectures such as the convolutional neural network (CNN) (LeCun and Bengio, 1995), the recurrent neural network (RNN) (Rodriguez et al., 1999), and the RNN with long short-term memory (LSTM) (Hochreiter and Schmidhuber, 1997).

To solve the ESI problem, deep learning frameworks have also been proposed in the past years, but with only a few existing works available. For example, Bore et al. (2021) introduced an RNN with LSTM units for spatiotemporal EEG source imaging and the proposed approach achieved good performance against the benchmark algorithms. Hecker et al. (2021) constructed a novel CNN-based structure, named ConvDip, to detect multiple sources, and this architecture is shown to outperform state-of-the-art methods. Wei et al. (2021) proposed an edge sparse basis network to learn the mapping between edge sparse source activation and recorded EEG signal.

As the source signal is defined on an irregular source space, where each source is defined as a vertex in a 3D source space (Liu et al., 2018), there exists a spatially connected graph structure among sources that have not been fully explored in the existing literature, especially with the recent advance of graph signal processing (Huang et al., 2016). In this work, we propose to employ the *spatial-temporal structure* of EEG source signal and come up with a new framework based on spatial graph Fourier transform (GFT) (Sandryhaila and Moura, 2013), and bidirectional LSTM (BiLSTM) neural network (Schuster and Paliwal, 1997), termed as GFT-BiLSTM to solve the ESI inverse problem. The main contributions of this paper are as follows:

(i) We propose to use the GFT on the 3D source space, and delineate the source space into spatially high, medium and low frequency subspaces spanned by corresponding eigenvectors, and the low frequency components naturally serve as a basis to estimate an extended areas of source activation.
(ii) By projecting the original source signal into a reduced dimensional subspace with low frequency eigenvectors, the dimension of output layer of BiLSTM can be greatly reduced.
(iii) The numerical experiments show that the proposed GFT-BiLSTM outperforms the benchmark algorithms based on area under the curve (AUC) and the localization error (LE).

## Related Work

Given the ill-posedness nature of ESI, traditional methods typically adopt parsimonious models to get a unique solution by introducing priors or regularizations based on the assumptions from neural physiology, brain anatomy, etc. (Scherg and Berg, 1991). The first category of ESI approaches is the equivalent current dipole (ECD) source localization (Cover et al., 2007). This method treats the neural electrical activity of the cerebral cortex as one of several ECDs. With such a constraint, the spatial location and orientation of each ECD can be optimized to best interpret the measured EEG signals. The ECD model has played a certain role in the localization of focal brain activity. However, the real brain sources can have multiple source activations (Sanei and Chambers, 2013), while the ECD method can only locate a single source point which makes it unable to reconstruct the distributed pattern of activated sources (Zumer et al., 2007). Another category of ESI methods, namely distributed source localization framework, has become more widely used in recent years. The current density distribution (CDD) model-based approach does not make any prior assumptions on the number of dipoles but divides the cerebral cortex into numerous triangular grids (Liao et al., 2012). The neural electrical activity on the brain voxels is represented by brain sources defined on the 3D mesh grid. Since the location of each source in the CDD model is fixed, the distributed source imaging only needs to solve a linear inverse problem (Astolfi et al., 2004). Over the past few decades, many distributed source imaging algorithms have been developed. The most popular ones are based on *L*_2_ norm constraints such as the minimum norm estimate (MNE) (Koles, 1998), the dynamic statistical parametric mapping (dSPM) (Tanaka et al., 2009), the low-resolution electromagnetic tomography analysis (LORETA), and the exact LORETA (eLORETA), (Jatoi et al., 2014), etc. The computation of these methods is simple, but the resulting solutions can be overdiffuse (Ou et al., 2009). Consequently, the sparsity constraints-based source imaging algorithms have been proposed by many researchers, such as the minimum current estimate (MCE) (Wen et al., 1998), the focal underdetermined system solver (FOCUSS) (Murray and Kreutz-Delgado, 2001), etc. Another class of methods for the ESI inverse problem is the data-driven method, which mainly includes the subspace-based classic Multiple Signal Classification (MUSIC) (Vergallo and Lay-Ekuakille, 2013) and the beamforming approaches, such as the linearly constrained minimum variance (LCMV) beamformer (Lin et al., 2008). MUSIC is the version of the Spatio-temporal approach. Multiple dipoles can be found in this technique *via* scanning potential locations through one dipole model (Mosher et al., 1992). The LCMV beamformer is a type of adaptive spatial filter that localizes activity sources by minimizing the contributions of other uncorrelated sources (Wong and Gordon, 2009). Recent developments on ESI include some interesting works such as utilizing more sophisticated edge-sparse regularization (Sohrabpour et al., 2020), or multitask framework for source localization among multiple subjects (Janati et al., 2020), or employing manifold graph structure in the EEG source space (Liu et al., 2021), source localization using multimodality of fMRI and EEG (Nguyen et al., 2018). However, the graph structure of the spatially connected sources is not fully explored in the literature, as the graph signal processing technique (Ortega et al., 2018) can have a principled way to decompose the spatial graph signal into components with different spatial frequencies. In this work, we come up with a new framework based on spatial graph Fourier transform and bidirectional LSTM (BiLSTM) neural network to efficiently solve the brain source extents reconstruction problem.

## Materials and Methods

In this section, we first give a brief introduction of the forward problem, then the spatial graph signal processing technique is explained, followed by structure of the BiLSTM neural network and finally, the GFT-BiLSTM model is introduced.

### Forward Problem

The relationship between the scalp potential measured by the electrodes and the brain source distribution can be expressed as follows:


(1) x(t)=Hs(t)+ε(t)

where *t* represents the time, vector **x**(*t*) ∈ **R**^*n* × 1^ represents the EEG or MEG signal measured by *n* electrodes, matrix **H** ∈ **R**^*n* × *m*^ represents the lead field, vector **s**(*t*) ∈ **R**^*m* × 1^ represents the source signal generated by *m* brain sources, and vector ε(*t*) ∈ **R**^*n* × 1^ represents the additive noise from observation.

The forward model models the linear mapping between scalp potential measured by the electrodes and the brain source signal (Birot et al., 2014). The solution to the forward problem relies on the establishment of the head model, which is determined by the geometry and corresponding electrical conductivity of different head tissues such as brain, skull, scalp, etc. (Acar and Makeig, 2010). In the early days, the mainly used head models are the spherical model and the ellipsoid model (Gutiérrez et al., 2005). With the development of brain imaging technology, real head models, which can be calculated by the boundary element method (BEM), the finite elements method (FEM), and the finite difference method (FDM) are increasingly used (Akalin-Acar and Gençer, 2004). Once the head model is established, the lead field matrix can be determined.

### Graph Signal Processing in the Brain Source Space

**FIGURE 1:**
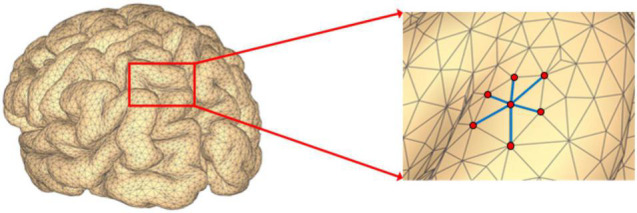
Illustration of brain mesh and brain source extent activation.

The connectivity relationship between *m* sources can be represented by an undirected graph, which can be defined as follows:


(2) G=(V,A)

where *V* ∈ **R**^*m* × 1^ is a set of *m* nodes, **A** ∈ **R**^*m* × *m*^ is the corresponding adjacency matrix. If there is no edge connecting nodes *i* and *j*, then *a*_*ij*_ = 0; otherwise, *a*_*ij*_ > 0, and its value represents the weight of the edge between the two nodes. In addition, since *G* is an undirected graph, then *a*_*ij*_ = *a*_*ji*_, which means the adjacency matrix ***A*** is symmetric. In the EEG source space, all the potential source locations are represented by the nodes defined on a 3D mesh as is illustrated in Figure 1. When the source locations *i* and *j* are neighbors on the 3D mesh, then we set *a*_*ij*_ > 0.

The graph signal is defined on the set of graph nodes *V*, which is represented by a vector, and each element represents the signal value at the corresponding node. The brain source signal **s** = [*s*_1_, *s*_2_, …, *s*_*m*_]^*T*^, in which each element *s*_*i*_ represents the signal value of the *i*-th source voxel, is defined on the nodes of graph *G*. The traditional Fourier transform calculates the projection of a function *f*(*t*) on the basis function *e*^−*iwt*^, and the projected value of a time series signal using Fourier transform *F*(*w*) represents its magnitude at the basis of a specific frequency. Different from the traditional Fourier transform defined in the temporal domain, for signals defined on a graph, the eigenvectors of the Laplacian matrix **L** of the graph can be used as the basis vectors of the GFT, where the Laplacian matrix **L** can be calculated as follows:


(3) L=D-A

where **L** ∈ **R**^*m* × *m*^, and **D** ∈ **R**^*m* × *m*^ is a diagonal matrix called the degree matrix, in which the diagonal elements satisfy $d_{ii}=\sum_{j}^{m}a_{ij}$, that is, the sum of elements in the *i*-th row of ***A***. Since ***A*** and ***L*** is real and symmetric, therefore, ***L*** can be decomposed as follows:


(4) $$\text{L}=\text{U}\Lambda {\text{U}}^{T}$$

where **Λ** ∈ **R**^*m* × *m*^ is a diagonal matrix and the diagonal elements λ_*i*_, (*i* = 1, 2, …, *m*) are the eigenvalues of ***L*** and satisfy λ_1_ ≤ λ_2_ ≤ … ≤ λ_*m*_, **U** ∈ **R**^*m* × *m*^ is the eigenvector matrix, which is also an orthogonal matrix satisfying **UU**^*T*^ = **I**, each column in **U** is an eigenvector of ***L*** and corresponds to the eigenvalue in **Λ**. With the eigenvectors of the Laplacian matrix ***L*** as the Fourier basis vectors, each eigenvector can be regarded as a graph basis with a certain frequency, and this frequency corresponds to the eigenvalue. The smaller the eigenvalue, the lower the frequency of the corresponding eigenvector, which is manifested as a small difference between the signals of adjacent nodes on the graph; on the contrary, a larger variation among neighboring signals. The value of a graph Fourier coefficient can measure the amplitude of the graph signal at different frequencies. With the eigenvectors as the Fourier basis vectors, the GFT of a given graph signal ***s*** can be defined as follows:


(5) $$\tilde{\text{s}}={\text{U}}^{T}\text{s}$$

where vector $\tilde{\text{s}}\in R^{m\times 1}$ is the graph Fourier coefficient. Further, the inverse graph Fourier transform (IGFT) of ***s*** can be defined as:


(6) $$\text{s}=\text{U}\tilde{\text{s}}$$

The above two formulas show that a graph signal can be decomposed into components with different frequencies through the GFT, and can also be recovered through the IGFT.

**FIGURE 2:**
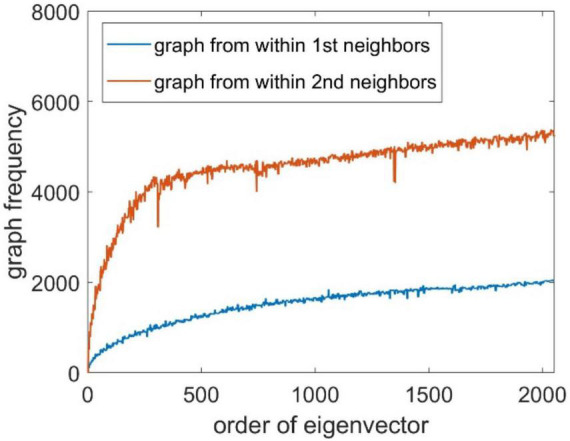
Graph frequency of the eigenvectors.

To characterize the graph frequency, we introduce the following definition:

**Definition 1:** Graph Frequency (GF): GF, denoted as *f*_*G*_, is a function of *u_i_* which represents the total number of sign flips of *u_i_* between any two connected nodes on *G*, it is defined as follows:


(7) $$f_{G}\left(u_{i}\right)=\sum_{j=1}^{m}\sum_{p\in \Omega \left(j\right)}I\left(u_{i}\left(j\right)u_{i}\left(p\right)<0\right)/2$$

where Ω(*j*) represents all neighbors of node *j*, and *I*(⋅) is an indicator function to check whether the values of *u_i_* at nodes *j* and *p* have a sign flip. The number of sign flips is analogous to counting the number of zero crossings of the basis signal within a given window for a time series data. We constructed the Laplacian matrices within first-order neighbors and second-order neighbors, and the associated GF spectrum is shown in Figure 2. It can be seen that the GF value of the eigenvector increases as the eigenvalue increases.

Similar to the counterpart in the time domain, the spatial frequency basis matrix **U** can be similarly decomposed into different spatial frequency bands, such as **U** = [**U**_*low*_, **U**_*medium*_, **U**_*high*_]. The graph signal ***s*** can be projected into a subspace of **U**. For example, $\tilde{\text{s}}={\text{U}}_{low}^{T}\text{s}$ is the projection of **s** into a space spanned by low frequency eigenvectors. In our work, we use the spatially low frequency components as a filter to reconstruct the focally extended sources.

### Bidirectional Long-Short Term Memory Neural Network

Bidirectional long-short term memory neural network is an extension of the traditional RNN (Schuster and Paliwal, 1997). For the time series, it is recognized that RNN can effectively estimate the information at the future moment based on the previous states. However, for the time series with a long sequence of states, the estimation performance of RNN will be greatly discounted, because the future information in a long time series usually depends on the information from distant history moments, which is the long-term dependence. However, the superior structure of the LSTM unit equips the network with the ability to solve long-term dependence. The RNN with LSTM units can filter the information by a unique structure called “gate” and store the valid information by the so-called “memory cell.” The elements in a gate vector have values in the interval [0,1]. When preceding time series information arrives at the gate, it will be multiplied with the gate vector element-wise, if the element value in the gate vector is 1, then the timing information multiplied with it will be retained, and if the element value is 0, the information will be discarded after a multiplication with 0. In this way, the filtering of information propagated in the LSTM unit is achieved. The valid information obtained after filtering is then stored in the memory cell and passed on to the next moment to prevent being lost over time, thus effectively addressing the long-term dependence existing in traditional RNNs.

**FIGURE 3:**
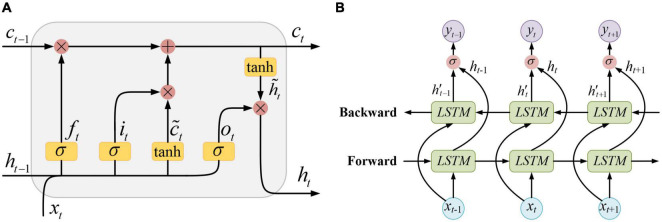
**(A)** The LSTM unit, **(B)** The BiLSTM network.

The structure of a standard LSTM unit is shown in Figure 3A, where **x**_*t*_, **h**_*t*−1_, and **c**_*t*−1_, respectively, represent the input sample at the current moment, the unit output and the memory state at the previous moment. The information contained in **x**_*t*_ and **h**_*t*−1_ is first activated by σ(⋅) function and got the forget gate **f**_*t*_, the input gate **i**_*t*_, and the output gate **o**_*t*_. At the same time, **x**_*t*_ and **h**_*t*−1_ are also activated by *tanh*(⋅) function and got a temporary state ${\tilde{\text{c}}}_{t}$. On the one hand, the information passed from the previous moment which contained in **c**_*t*−1_ is filtered by the forget gate **f**_*t*_. On the other hand, the newly input information contained in ${\tilde{\text{c}}}_{t}$ is filtered by the input gate **i**_*t*_. Then, the valid information retained by the above two filtering processes is integrated together as a new memory state **c**_*t*_. This newly updated memory state is passed along time to the next moment, and simultaneously, it is also filtered by the output gate **o**_*t*_. Finally, the new output of the LSTM unit **h**_*t*_ is obtained.

This propagation process can be formulated as follows:


(8) $${\text{f}}_{t}=\sigma \left({\text{W}}_{f}\left[{\text{h}}_{t-1},{\text{x}}_{t}\right]+{\text{b}}_{f}\right)$$

(9) $${\text{i}}_{t}=\sigma \left({\text{W}}_{i}\left[{\text{h}}_{t-1},{\text{x}}_{t}\right]+{\text{b}}_{i}\right)$$

(10) $${\text{c}}_{t}=tanh\left({\text{W}}_{c}\left[{\text{h}}_{t-1},{\text{x}}_{t}\right]+{\text{b}}_{c}\right)$$

(11) $${\text{c}}_{t}={\text{f}}_{t}*{\text{c}}_{t-1}+{\text{i}}_{t}*{\tilde{\text{c}}}_{t}$$

(12) $${\text{o}}_{t}=\sigma \left({\text{W}}_{o}\left[{\text{h}}_{t-1},{\text{x}}_{t}\right]+{\text{b}}_{o}\right)$$

(13) $${\text{h}}_{t}={\text{o}}_{t}*\text{tanh}\left({\text{c}}_{t}\right)$$

where **W**_*f*_, **W**_*i*_, **W**_*c*_, **W**_*o*_ are weight matrices; **b**_*f*_, **b**_*i*_, **b**_*c*_, **b**_*o*_ are bias vectors; the symbol * stands for the element-wise multiplication.

The hidden layer of the BiLSTM neural network is composed of two layers of LSTM units that are reversely connected, and its structure is shown in Figure 3B. In a BiLSTM layer, the time series performs both forward propagation and backward propagation. Therefore, both the information at the previous moments and that at the future moments can be fully utilized.

The output of the BiLSTM neural network can be calculated as follows:


(14) $${\text{y}}_{t}=\sigma \left({\text{W}}_{s}\left[{\text{h}}_{t}⊕{\text{h}}_{t}^{\prime }\right]+{\text{b}}_{s}\right)$$

where **y**_*t*_ is the final output, ${\text{h}}_{t}^{\prime }$ is the unit output of the backward propagation, **W**_*s*_ and **b**_*s*_ are the weight matrix and the bias vector of the output layer, respectively, and the symbol ⊕ stands for the vector concatenation.

### Graph Fourier Transform-Bidirectional Long-Short Term Memory for Electrophysiological Source Imaging

#### Graph Fourier Transform-Bidirectional Long-Short Term Memory Training Procedure

Generally, the brain is divided into smaller voxels, and each voxel can be activated and regarded as a source. Therefore, when a BiLSTM network is adopted to solve the inverse problem of ESI with the recorded EEG signal as the inputs and the source signal as the outputs, the number of nodes in the output layer of the network equals to the number of sources. This will lead to a significant number of parameters in the network. In order to improve the training speed of the BiLSTM network, in this paper, we reduce its output nodes by using projected coefficients as the output dimension based on low frequency eigenvectors, given the extended source activation pattern mainly contains signal from the low frequency subspace. With the training dataset {**x**_*i*_, **s**_*i*_}, the training setup procedure is as follows:

Step 1: Perform the GFT on the original brain source signal ***s*** according to (5), then the Fourier coefficient ***s̃*** is obtained.

Step 2: With the eigenvectors in ***U*** as the Fourier basis vectors, and the corresponding eigenvalues in ***Λ***. Then, set the eigenvalue threshold as ***T*_*f*_**, and the number of eigenvalues less than ***T*_*f*_** is ***k***.

Step 3: Take the first ***k*** columns of eigenvectors in ***U***, denoted as ***U*_*k*_** and the first ***k*** elements in the Fourier coefficients ***s̃*** as ***s̃′***.

Step 4: Set the number of the input nodes in the BiLSTM network as ***n***, the number of the output nodes as ***k***, and the number of the BiLSTM units in the hidden layer as ***l***. Then take the EEG signal ***x*** as the input, ***s̃′*** as the output to train the BiLSTM network.

The mean square error (MSE) is chosen as the loss function:


(15) $$MSE=\frac{1}{N}\sum_{i=1}^{N}{\left({\tilde{\text{s}}}_{i}^{\prime }-{\hat{\text{s}}}_{i}^{\prime }\right)}^{2}$$

where *N* is the number of data points, ${\tilde{\text{s}}}_{i}^{\prime }$ is the true values, and ${\hat{\text{s}}}_{i}^{\prime }$ is the estimated values by the network. The Nadam optimizer is adopted during the training process.

In general, we take the projections of the brain source signal on the basis spanned by the low frequency eigenvectors instead of the brain source signal itself as the output of the BiLSTM network. By doing this, the number of output nodes in the network can be reduced from *m* to *k*, which can make the parameters in the network significantly decrease and the training speed increase. In the meanwhile, the source extent pattern is recovered better after removing the spatial high frequency noise.

#### Source Signal Recovery

**FIGURE 4:**
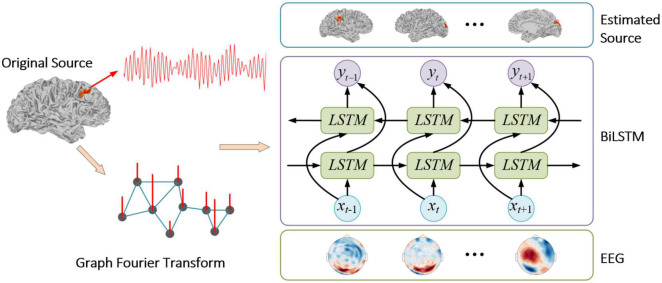
The flowchart for the proposed method.

When the network training is completed, perform the IGFT on the estimated values $\hat{\text{s}}$ as follows:


(16) $$\hat{\text{s}}={\text{U}}_{k}{\hat{\text{s}}}^{\prime }$$

where $\hat{\text{s}}$ is the estimated source signal. The whole process is summarized in Figure 4.

## Experiments

In this section, the proposed GFT-BiLSTM is evaluated using both the synthetic data and the real data. The benchmark ESI algorithms, including dSPM, MNE, and sLORETA, are used for comparison.

In the simulated data, the number of brain sources is 2,052 and the number of electrodes is 128, then these source regions are activated in turn with one level neighborhood sources (sources that are directly connected to activated source in 3D mesh) activated at the same time, and the source signal time series is generated based on the 5th-order autoregressive (AR) model (Haufe and Ewald, 2019), with 100 Hz sampling rate and 1 s of length, and the simulated source signal *s* is obtained. Given the lead field matrix *H*, by using the forward model, the EEG data ***x*** can be calculated according to Eq. (1), in which the sensor noise ε is generated based on different signal-to-noise ratio (SNR) levels (20 dB, 30 dB, and 40 dB), where SNR is defined based on the ratio of the power of signal *P*_*signal*_ to the power of noise *P*_*noise*_, as prescribed below:


(17) $$SNR=10log\left(\frac{P_{signal}}{P_{noise}}\right)$$

The Laplacian matrix ***L*** of the brain source signal is calculated according to Eq. 3, then decomposed according to Eq. 4 to obtain the eigenvalue matrix *****Λ***** and the corresponding eigenvector matrix ***U***. Use the eigenvectors as the basis vectors of the GFT, then the Fourier coefficient is obtained according to Eq. 5. We set *k* = 615 as the number of eigenvectors, based on the frequency spectrum illustrate in Figure 2. We use the first *k* values of $\tilde{\text{s}}$ as the model output ${\tilde{\text{s}}}^{\prime }$, and the EEG data is taken as the model input. The simulated data is divided into training, validation, and testing datasets according to the proportion of 70%, 15%, and 15%, respectively. The number of input nodes in the BiLSTM neural network is set to be 128, the hidden nodes is set to be 2,560, and the number of output nodes is 615. Adopt the MSE as the loss function and Nadam optimizer to train the BiLSTM neural network on the training set. After training, the testing dataset is used for model testing. The following two metrics are used as the metrics for model evaluation:

• Localization error: LE can be quantified as the distance between the true peak source point and estimated peak source point.
• Area under the curve: AUC measures the area underneath the receiver operating characteristic (ROC) curve.

### Evaluation With Single Source

**FIGURE 5:**
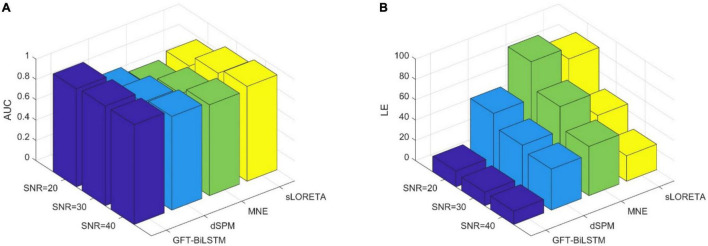
The performance comparison of different ESI inverse solutions with a single activated area. **(A)** The comparison of AUC at different SNR levels. **(B)** The comparison of LE at different SNR levels.

**FIGURE 6:**
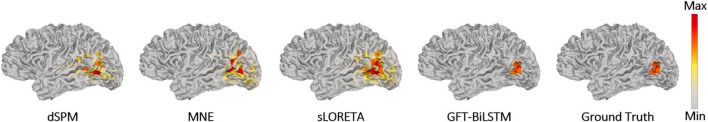
Brain source activations estimated by different ESI algorithms with a single activated area.

To render source extents activation, the adjacent sources along with a central source are activated at the same time. The signal strength of the adjacent sources is set to be lower than that of the central region. All the 2,052 potential source locations are chosen as the central source in turn to generate the scalp EEG data. In the first experiment, we test the proposed algorithm on the simulated EEG data and the true source activation pattern with one source extents activated. Apply the training and validation dataset to train and validate the proposed GFT-BiLSTM model, and then test it on the testing dataset. The performance of the GFT-BiLSTM is compared with that of dSPM, MNE, and sLORETA. The evaluation metrics of each model are shown in Table 1 and Figure 5. The comparison between the ground truth source and the reconstructed sources by different algorithms is shown in Figure 6.

**TABLE 1:** The evaluation metrics corresponding to different ESI inverse solutions with a single activated area.

	AUC			LE		
	SNR = 20	SNR = 30	SNR = 40	SNR = 20	SNR = 30	SNR = 40
GFT-BiLSTM	0.9668	0.9821	0.9844	15.5683	13.2668	13.2067
dSPM	0.7733	0.8769	0.9237	58.1761	45.4715	40.3180
MNE	0.7020	0.8192	0.8954	94.8286	69.2218	48.5848
sLORETA	0.7637	0.8784	0.9339	83.3185	46.3218	25.1723
						

From Table 1 and Figure 5, it can be seen that the proposed GFT-BiLSTM shows the better performance when compared to other methods. For different SNR levels, the AUC corresponding to GFT-BiLSTM is the highest while the LE is the lowest. The brain source distribution estimated by the proposed GFT-BiLSTM is closer to the ground truth as illustrated in Figure 6. The numerical result demonstrates the superiority of the GFT-BiLSTM when applied to solve the ESI inverse problem.

### Evaluation With Multiple Sources

**FIGURE 7:**
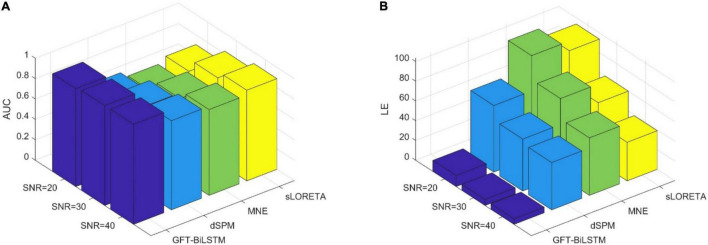
The performance metrics comparison of different ESI inverse solutions with multiple activated areas. **(A)** The comparison of AUC at different SNR levels. **(B)** The comparison of LE at different SNR levels.

**FIGURE 8:**
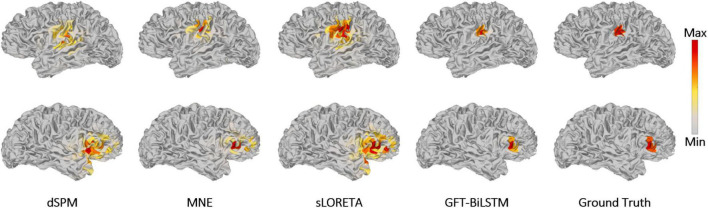
Brain source activation reconstructed by different ESI algorithms with multiple activated areas. The upper figures correspond to the activated area in the left side of the brain, the bottom figures correspond to the activated area in the right side of the brain.

In order to study the performance of the proposed GFT-BiLSTM when there are multiple activated sources, we randomly select 2 out of 2,052 brain source locations to be activated and the first level neighboring sources of these two source locations are also activated with a lower signal magnitude. Apply this simulated dataset to train and validate the GFT-BiLSTM, and then test it on the testing dataset. The performance of the GFT-BiLSTM is compared with that of dSPM, MNE, and sLORETA. The evaluation metrics of each model are shown in Table 2 and Figure 7. The comparison between the real source and the estimated source is shown in Figure 8.

**TABLE 2:** The evaluation metrics corresponding to different ESI inverse solutions with multiple activated areas.

	AUC			LE		
	SNR = 20	SNR = 30	SNR = 40	SNR = 20	SNR = 30	SNR = 40
GFT-BiLSTM	0.9602	0.9796	0.9818	11.3105	6.1145	5.6589
dSPM	0.7130	0.8214	0.8743	67.4522	52.6055	47.9739
MNE	0.6523	0.7640	0.8415	103.3403	79.0106	58.2711
sLORETA	0.7045	0.8253	0.8892	93.6530	60.1024	39.0890
						

From Table 2 and Figure 7, the proposed GFT-BiLSTM demonstrates better performance when compared with other methods. In addition, with the increased number of activated source, the estimation performance of all methods except the GFT-BiLSTM deteriorates significantly. This is demonstrated as an increase in LE and a decreased AUC value. There is also a situation for other method in which the localization of the central region is accurate while the localization of its adjacent regions is slightly deviated. The reason is that as the number of activated regions increases, the distribution of the sources is no longer concentrated, and it is more challenging to accurately estimate the locations of all active regions. The reduction in performance for AUC is much more pronounced for the benchmark algorithms. In contrast, the performance of the GFT-BiLSTM is more stable and robust when it comes to multiple activated sources.

### Evaluation With Real Epilepsy Data

**FIGURE 9:**
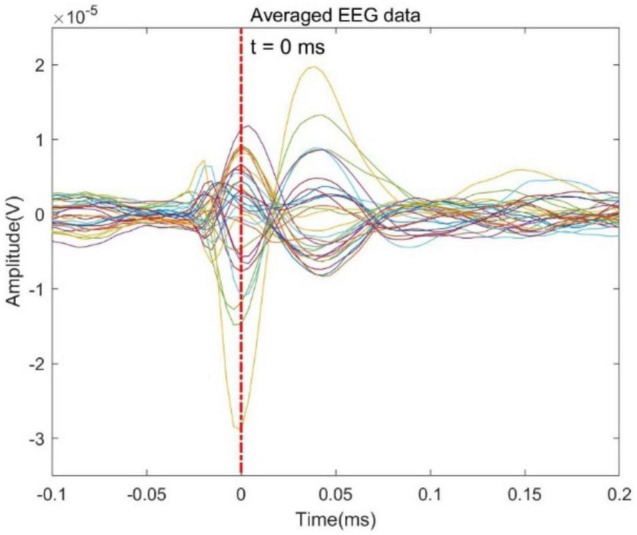
Average EEG time series plot around the inter-ictal spike.

**FIGURE 10:**
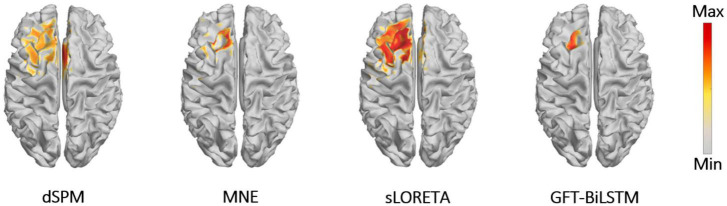
Reconstructed sources by different ESI algorithms for epilepsy EEG data.

In order to further evaluate the performance of the proposed GFT-BiLSTM, we applied it on the public epilepsy EEG dataset from the Brainstorm tutorial datasets (Tadel et al., 2011). This dataset was recorded from a patient who suffered from focal epilepsy. The patient underwent invasive EEG to identify the epileptogenic area then underwent a left frontal tailored resection and was seizure-free during a 5-year follow-up period. We followed the Brainstorm tutorial to obtain the head model, and the lead field matrix. Then we calculated the average spikes (as shown in Figure 9) of the provided EEG measurements with 29 channels. Apply the averaged EEG data for brain source localization, and the estimated sources at peak (0 ms) from different methods are shown in Figure 10, as compared to other methods including dSPM, MNE, sLORETA.

It can be seen from Figure 10 that the proposed GFT-BiLSTM provides a good reconstruction of the epileptogenic zone which was validated by the follow-up survey after resection on the left frontal region. The source area estimated by dSPM and sLORETA spans a wide range cortical areas and includes part of the right frontal lobe which is not related to the epilepsy lesion. In contrast, the source location estimated by the MNE method and the GFT-BiLSTM proposed in this paper is more accurate. However, compared between the two methods, the range of sources estimated by the GFT-BiLSTM is smaller, and the source estimated by GFT-BiLSTM shows better continuity of the spatial signal, due to the benefit of using GFT.

## Conclusion

The inverse problem of source extents reconstruction is challenging due to its highly ill-posed nature. In this paper, we present a novel ESI framework, named GFT-BiLSTM, which is based on the delineation of spatial graph frequency using graph Fourier transform and BiLSTM, to solve the ESI problem in a more efficient and robust way. Our numerical results based on the synthetic data and real data show that the proposed GFT-BiLSTM has a superior performance compared to other benchmark methods. The future work can further explore more clinical applications using the proposed framework. A more rigorous selection of the low frequency set of eigenvectors can also be investigated.

## Data Availability Statement

The original contributions presented in the study are included in the article/supplementary material, further inquiries can be directed to the corresponding author.

## Author Contributions

MJ: methodology, software, investigation, data curation, and writing – original draft. GW: methodology, conceptualization, writing, review, and editing. YG: methodology and writing – original draft. DW and HL: methodology, writing, review, and editing. JX: conceptualization, writing, review, and editing. FL: conceptualization, methodology, writing – original draft, review, editing, and supervision. All authors contributed to the article and approved the submitted version.

## Conflict of Interest

The authors declare that the research was conducted in the absence of any commercial or financial relationships that could be construed as a potential conflict of interest.

## Publisher’s Note

All claims expressed in this article are solely those of the authors and do not necessarily represent those of their affiliated organizations, or those of the publisher, the editors and the reviewers. Any product that may be evaluated in this article, or claim that may be made by its manufacturer, is not guaranteed or endorsed by the publisher.
